# A modified graft‐versus‐host‐induced model for systemic sclerosis, with pulmonary fibrosis in Rag2‐deficient mice

**DOI:** 10.1002/2211-5463.12268

**Published:** 2017-08-16

**Authors:** Xue Yang, Chi Liu, Masayuki Fujino, Ji Yang, Xiao‐Kang Li, Hejian Zou

**Affiliations:** ^1^ Division of Rheumatology Huashan Hospital Fudan University Shanghai China; ^2^ Division of Transplantation Immunology National Research Institute for Child Health and Development Tokyo Japan; ^3^ Institute of Rheumatology, Immunology and Allergy Fudan University Shanghai China; ^4^ AIDS Research Center National Institute of Infectious Diseases Tokyo Japan; ^5^ Department of Dermatology Zhongshan Hospital Fudan University Shanghai China

**Keywords:** fibrosis, graft‐versus‐host disease, systemic sclerosis

## Abstract

Systemic sclerosis (SSc) is a connective tissue disease that results in fibrosis in multiple organs. Various animal models for this disease have been developed, both genetic and induced. One of the induced models, sclerodermatous graft‐versus‐host disease (scl‐GvHD), exhibits the main characteristics of SSc, but involves lethal γ‐irradiation of recipients. We sought to develop a modified scl‐GvHD model. Spleen cells from B10.D2 donor mice were transplanted into immunodeficient Rag‐2 recipients on the BALB/c genetic background. Tissue fibrosis was analyzed at 3 and 9 weeks after transplantation. In addition to serum levels of anti‐Scl‐70 autoantibody and cytokines, tissue inflammation, fibrosis, expression of collagen‐I and α‐smooth muscle actin (α‐SMA), infiltration of leukocytes, mRNA expression of transforming growth factor (TGF)‐β, collagen‐I, α‐SMA, tumor necrosis factor (TNF)‐α, and interleukin (IL)‐6, the classical signal pathway of TGF‐β, Smad‐3, and p‐Smad‐3 expression in tissue were analyzed. Skin thickening and increased collagen synthesis, as well as the manifestation of tissue fibrosis, could be detected in skin, kidney, and lung of modified scl‐GvHD mouse model. Increased serum levels of anti‐Scl‐70 autoantibody, IL‐10, and TGF‐β could be detected. Increased CD4^+^ T cells and F4/80^+^ macrophage infiltration were found in skin, kidney, and lung. Gene expression of collagen‐I, TGF‐β, α‐SMA, TNF‐α, and IL‐6 was increased in tissue of the scl‐GvHD model. Moreover, TGF‐β expression and Smad‐3 phosphorylation were detected in skin, kidney, and lung of scl‐GvHD mice. Our data show that spleen cells from B10.D2 donor mice transplanted into immunodeficient Rag‐2 recipients could induce typical fibrosis not only of the skin and kidney but also of lung, which was missing from previous scl‐GvHD models. Thus, the modified scl‐GvHD model might be a promising model to explore the immunologic mechanisms of SSc and may be useful for investigation of new therapies for systemic sclerosis.

**Abbreviations**
α‐SMA: α‐smooth muscle actin
IL: interleukin
qRT‐PCR: Quantitative reverse transcription‐polymerase chain reaction
Rag‐2: recombination‐activating gene 2
scl‐GvHD: sclerodermatous graft‐versus‐host disease
SSc: systemic sclerosis
TGF‐β: transforming growth factor‐β
TNF‐α: tumor necrosis factor‐α

Systemic sclerosis (SSc) is a connective tissue disease characterized by increased collagen synthesis and deposition, skin thickening, fibrosis of organs including the liver, kidney, intestinal tract, and lung, and widespread vasculopathy. SSc is distinctly characterized by the progressive interstitial and perivascular fibrosis 1. SSc is considered incurable. In particular, diffuse fibrosis and the interstitial pneumonia after infection are the biggest highest risks of fatality in SSc.

Although SSc has been studied intensively, the initial pathogenesis remains unclear. Viruses, drugs, environmental, and occupational exposures to organic solvents, vinyl chloride, and silicate have been considered to be the etiology of SSc 2. Furthermore, immune dysfunction is known to contribute to the autoimmune injuries in SSc 2, 3, 4, 5.

To elucidate the pathogenesis of SSc for the development of new therapeutic approaches and medicine, an animal model that can comprehensively mimic the disease is needed. This model should have the typical characteristics of SSc, including dermal thickening, progressive fibrosis of internal organs, vascularity markers, and autoantibodies. To date, several genetic and inducible animal models of SSc have been developed and are available for research 6. Animal models for SSc can be classified into two types. The first is genetic models, which mainly include Cre‐ER and TBRIIΔk mice, Fra‐2 transgenic mice, tight skin‐1 and ‐2 mouse models, and UCD‐200 chicken. The second is inducible models, which include topoisomerase‐I‐ and CFA‐induced fibrosis, hypochlorous acid‐induced fibrosis, bleomycin‐induced fibrosis, and sclerodermatous graft‐versus‐host disease (scl‐GvHD) 7. Each type of animal model has its own advantages and limitations 8. Among these animal models, scl‐GvHD model could comprehensively exhibit the three main features of the disease, namely vasculopathy, immune dysregulation, and dermal and internal organ fibrosis 1.

scl‐GvHD represents a distinctive phenotype of chronic GvHD first described in 1977 9. The scl‐GvHD model is considered to be one of the best for SSc studies because it can mimic most of the characteristics of SSc, including dermal thickening in extremities, overproduction of collagen, circulating autoantibodies, and internal organ fibrosis. Traditional scl‐GvHD was carried out by the transplantation of bone marrow and spleen cells from B10.D2 donor into BALB/c recipient mice following lethal γ‐irradiation of recipients 10. Two weeks after bone marrow and spleen cells transplantation, inflammatory cells infiltrate affected tissues and stimulate resident fibroblasts to release large amounts of collagen, resulting in tissue fibrosis. Notably, in the synthesis of an scl‐GvHD model, lethal γ‐irradiation of recipients always leads to death. Therefore, a stable, convenient, and feasible scl‐GvHD mouse model is needed. In this study, we created a modified model of scl‐GvHD using immunodeficient recombination‐activating gene 2 (Rag‐2) recipient mice.

## Materials and methods

### Animals

Female normal BALB/c (H2^d^) mice and B10.D2 mice (H2^d^) were purchased from Japan SLC, Inc (Shizuoka, Japan). Rag‐2‐KO mice on the BALB/c (H2^d^) background were kindly obtained from S. Nakae (The University of Tokyo). All mice were enrolled between 10 and 12 weeks of age and were maintained under standard conditions. Rodent food, water, and all animal manipulations were performed according to the recommendations of the Committee of the Care and Use of Laboratory Animals at the National Research Institute for Child Health and Development, Tokyo, Japan (Permission Number: 2002‐003).

### Scl‐GvHD model

Spleens were aseptically collected from BALB/c and B10.D2 mice separately. A single‐cell suspension was generated from dissociated spleen by rubbing. Erythrocytes were lysed in 10 × PBS and pure water, and living cells were counted by trypan blue dye exclusion assay on a cytometer. The splenocytes were resuspended in RPMI 1640 (Gibco BRL, Grand Island, NY, USA) and were purified by density gradient centrifugation using lympholyte‐M (Cedarlane Ltd, Burlington, ON, Canada). Cells were passed via a 75‐μm nylon mesh cell filter to obviate large debris. Splenocytes (4 × 10^7^) collected from B10.D2 mice (scl‐GvHD group) or BALB/c mice (control group) were transplanted intravenously into recipient Rag‐2‐KO mice.

### Histological analysis

Hematoxylin and eosin (H&E) staining, standard Masson trichrome histochemical staining, immunofluorescence analysis for collagen‐I deposition and α‐smooth muscle actin (α‐SMA) expression, and immunohistochemical analysis for the measurement of CD4^+^ T lymphocytes and F4/80^+^ macrophages infiltration were performed as previously reported 11, 12, 13. Histological dermal thickness, lung fibrosis, and hydroxyproline contents were determined as described previously 14.

### ELISA

Concentration of anti‐Scl‐70 autoantibody in serum was determined by ELISA (IBL, Gunma, Japan). Interleukin (IL)‐10 and TGF‐β in serum were also detected by ELISA (R&D, Minneapolis, MN, USA). The process of ELISA was performed by the manufacturer's instruction.

### Quantitative reverse transcription‐PCR (qRT‐PCR) analysis

RNA extraction from tissue and qRT‐PCR analysis were performed as described previously 15.

### Western blot

The western blot was performed as previously described. Primary antibodies used for this analysis were as follows: GAPDH (1 : 4000; Cell Signaling Technology, Beverly, MA, USA), Smad‐3, p‐Smad‐3 (1 : 1000; Cell Signaling Technology), TGF‐β (1 : 1000; Abcam plc, Cambridge, UK), and visualized using Image Quant LAS 4000 (GE Healthcare, Little Chalfont, UK).

### Statistical analysis

Results were expressed as means ± SD. Statistical significance was determined by ANOVA for comparisons of multiple means, Student's *t*‐test, or Mann–Whitney *U*‐test. Correlations were determined by Spearman's ranking. *P* < 0.05 was considered statistically significant.

## Results

### Skin fibrosis in Rag‐2‐KO mice induced by intravenous injection of B10.D2 mouse‐derived splenocytes

The skin is the most frequently involved organ in SSc, which mainly exhibits collagen overproduction and fibrosis 16. In our study, 4 × 10^7^ splenocytes derived from B10.D2 mouse were injected intravenously into recipient BALB/c Rag‐2‐KO mice, and splenocytes derived from BALB/c mouse were injected for the control group.

**Figure 1:**
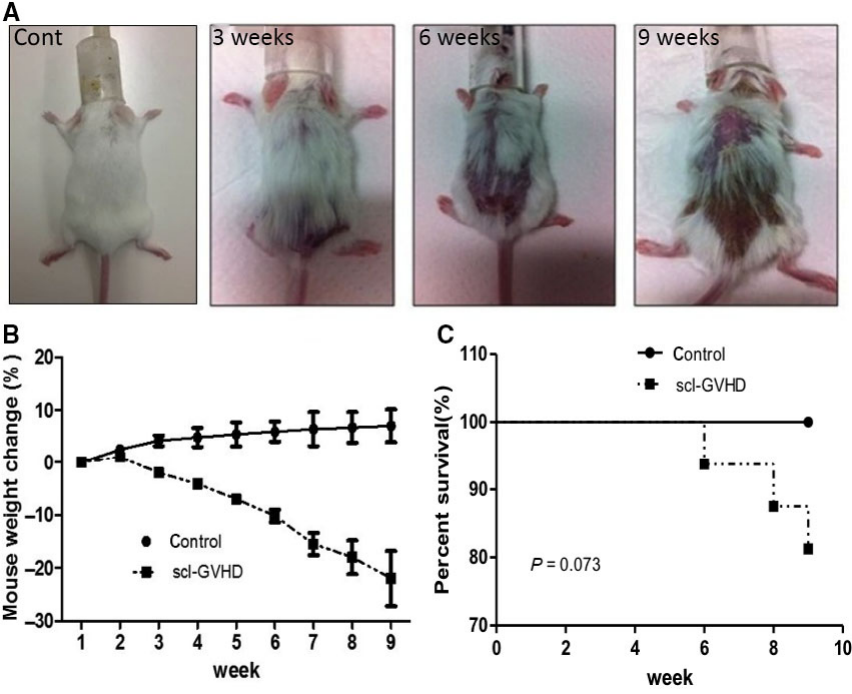
Skin injuries, body weight changes, and survival in Rag‐2‐KO mice induced by intravenous injection of B10.D2 mouse‐derived splenocytes. (A) Skin sclerosis and hair loss involvement of the scl‐GvHD mice at 3, 6, and 9 weeks after transplantation. (B) The weight changes in scl‐GvHD mice and control mice. (C) The survival rate of scl‐GvHD mice and control mice; there is no statistical significance between two groups (*P* = 0.073). Ten mice were used in this experiment (control, *n* = 4; scl‐GvHD, *n* = 6).

Our result showed that experimental scl‐GvHD mice developed skin thickening and hardening with hair loss 3 weeks after injection with B10.D2 mouse‐derived splenocytes. The involved area of skin sclerosis gradually increased, and serious skin sclerosis and hair loss were detected 9 weeks after transplantation (Fig. 1A). Meanwhile, the mice weight decreased with the progress of the disease (Fig. 1B). scl‐GvHD mice began to die from 6 week after injection with 4 × 10^7^ splenocytes derived from B10.D2 mouse; about 20% Scl‐GvHD mice died at 9 weeks after transplantation (Fig. 1C). The control group injected with splenocytes derived from BALB/c mouse did not show any skin sclerosis, hair loss, and death, but there was no statistical significance between two groups in terms of survival rate.

**Figure 2:**
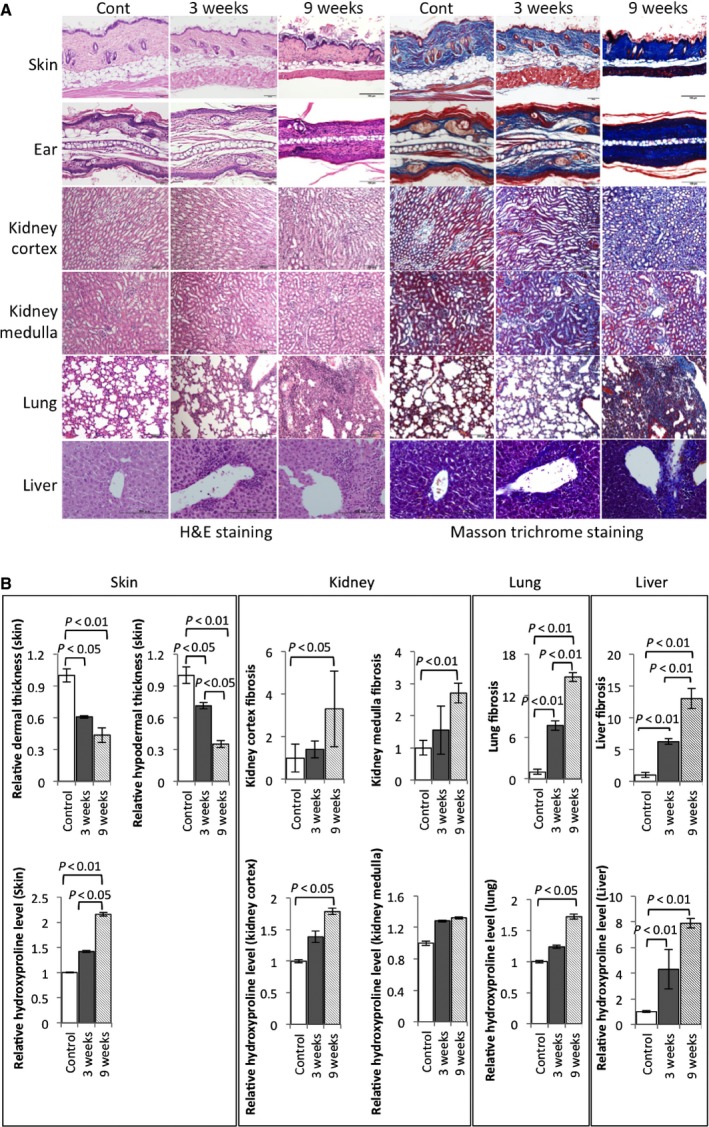
Tissue inflammation, fibrosis, extensive fibrosis, and collagen deposition in scl‐GvHD mice. (A) H&E staining and Masson's trichrome staining of upper back skin, ear, kidney (cortex, medulla), lung, and liver in control mice and scl‐GvHD mice after splenocytes transplantation of 3 and 9 weeks. Scale bars represent 100 μm. (B) The results of the quantification of relative dermal and hypodermal layers thickness in skin, lung, and liver fibrosis, relative hydroxyproline content in skin, kidney, lung, and liver. Data are the means ± SEM (*n* = 3–5). *P* < 0.05, *P* < 0.01 compared with the corresponding value of the control. Twelve mice were used in this experiment (control, *n* = 4; scl‐GvHD, *n* = 8).

Histological examination revealed marked dermal and subcutaneous tissue sclerosis, inflammatory cells infiltration, and skin appendages and fat tissue atrophy in upper back skin of scl‐GvHD mice (Fig. 2A: left 1st row and Fig. 2B: upper row, left column). Masson's trichrome staining further proved significant collagen deposition from 3 weeks after transplantation (Fig. 2A: right 1st row, 2nd column and Fig. 2B: lower row, left column). Dermal thickening declined because of skin appendages and fat tissue atrophy. More collagen deposition and connective tissue accumulation could be detected at 9 weeks (Fig. 2A: right 1st row, 3rd column and Fig. 2B: lower row, left column). Skin dermal sclerosis and collagen deposition are the characteristics of SSc. These data significantly proved that skin appendages, fat tissue atrophy, and collagen deposition in dermal and subcutaneous tissues could be induced in Rag‐2‐KO mice by intravenous injection of B10.D2 mouse‐derived splenocytes.

### Extensive fibrosis in Rag‐2‐KO mice induced by intravenous injection of B10.D2 mouse‐derived splenocytes

**Figure 3:**
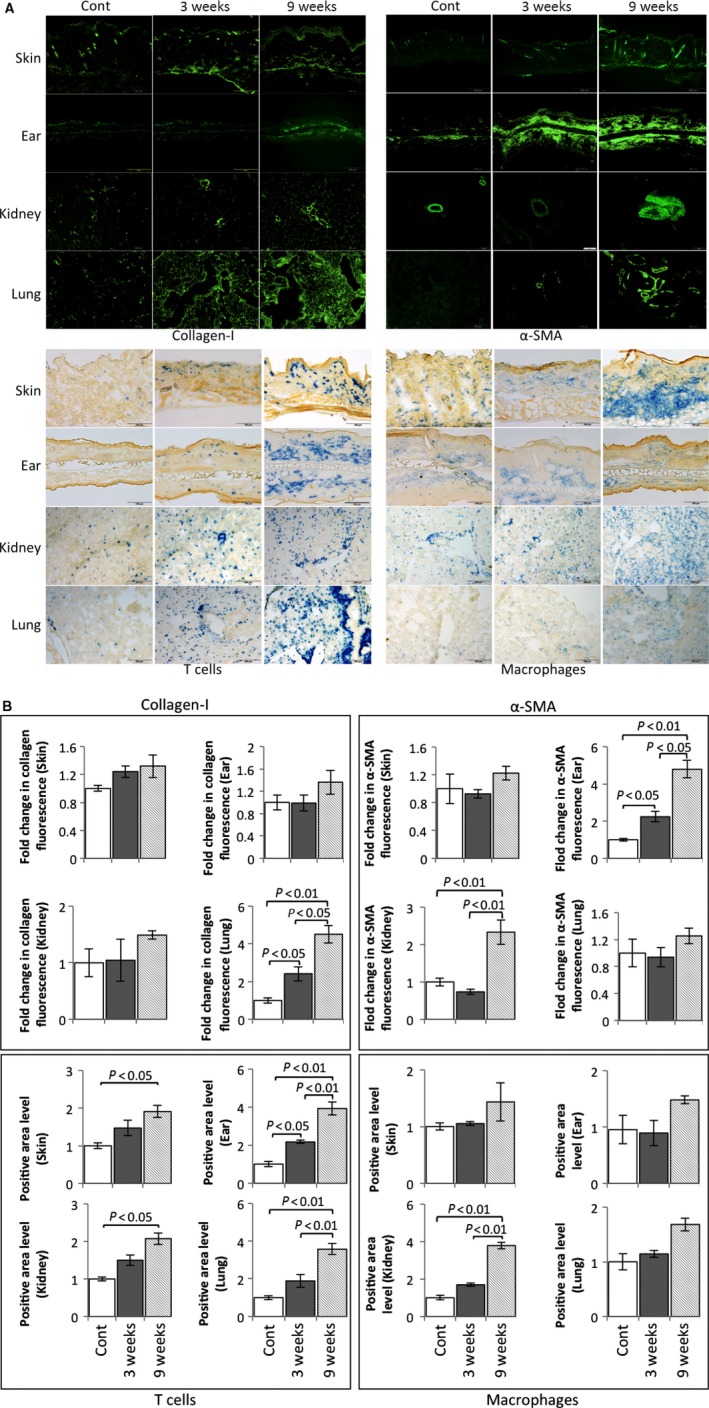
Collagen‐I and α‐SMA expression, T cells, and macrophages in scl‐GvHD mice. (A) Collagen‐I expression in upper back skin, ear, kidney, and lung tissues of control and scl‐GvHD mice after splenocytes transplantation of 3 and 9 weeks. Scale bars represent 100 μm. (B) The results of the quantification of fold change in collagen, α‐SMA, T cells, and macrophages in skin, ear, kidney, and lung. Data are the means ± SEM (*n* = 3–5). *P* < 0.05, *P* < 0.01 compared with the corresponding value of the control. Twelve mice were used in this experiment (control, *n* = 4; scl‐GvHD, *n* = 8).

In a systemic model for SSc, dermal thickening and collagen deposition were major manifestations, and in this study, these symptoms were found in skin as well as in ear (Fig. 2A: 2nd row). In addition, SSc has extensive involvement, such as intestinal tract, kidney, liver, and lung. Using HE and Masson's trichrome staining, in our scl‐GvHD model, collagen overproduction and increased infiltration of inflammatory cells were detected not only in liver, kidney cortex and medulla but also in lung tissue (Fig. 2A: 3rd, 4th, 5th, and 6th row, and Fig. 2B). Furthermore, our immunofluorescence data showed that overexpression of collagen‐I also could be detected in skin, kidney, but the most significantly increased in lung tissue (Fig. 3A, upper left and 3B).

Immunofluorescence staining for the detection of α‐SMA expression was performed to characterize additively the vascular changes in the murine scl‐GvHD model. As shown in Fig. 3A (upper right) and 3B, α‐SMA mainly expresses on activated fibroblasts and more α‐SMA‐positive cells were found in ear skin and lung tissue of scl‐GVHD mice. All these data proved that fibrosis not only involved the skin but also internal organs, especially lung tissue in our scl‐GvHD mice.

### Immune cells infiltration, autoantibody, and inflammatory cytokines expression are increased in Scl‐GvHD model

Complex inflammatory cell infiltration in the skin is the symptom of scl‐GvHD model. Our data showed increased CD4^+^ T lymphocytes and F4/80^+^ macrophages infiltration in upper back skin, ear, kidney, and lung with the progress of disease activity (Fig. 3A, lower and 3B). These data indicate that immune cells infiltration was involved in the course of scl‐GvHD model; the scl‐GvHD model has the characteristics of immune cell dysfunction.

**Figure 4:**
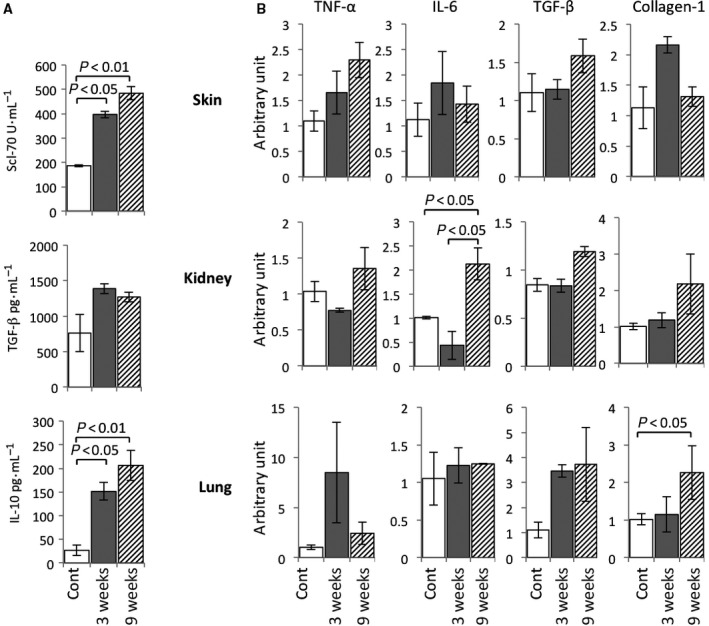
Autoantibody and inflammatory cytokines related to fibrosis expression, and the gene expression of collagen synthesis and deposition, vascular change, inflammatory factors in scl‐GvHD mice. (A) Serum amounts of the Scl‐70 antibody, TGF‐β, and IL‐10 in scl‐GvHD mice at 3 and 9 weeks. The data shown are the means ± SD. *P* < 0.05, *P* < 0.01 compared with the corresponding value of the control; *n* = 6 in each group. (B) The mRNA expression of TNF‐α, IL‐6, TGF‐β, and collagen‐I genes in skin, kidney, and lung of the control and scl‐GvHD mice after splenocytes transplantation of 3 and 9 weeks. The relative quantity is presented as the ratio of the comparative cycle threshold (Ct) of the target genes against those of the housekeeping gene, 18S. Data are representative of three independent experiments and indicate the mean ratio of triplicate results from each experiment. Data are the means ± SEM (*n* = 3–5).

Anti‐topoisomerase antibody I (Scl‐70) is known as a hallmark antibody of SSc 1. We found that the serum concentration of anti‐Scl‐70 antibody was increased in the scl‐GvHD model compared with that in the control group (Fig. 4A). Furthermore, we examined the fibrosis‐related cytokines in our scl‐GVHD model. Our data showed that the serum concentration of IL‐10 was significantly increased and TGF‐β had the trend of elevation in scl‐GVHD model (Fig. 4A).

Meanwhile, we also detected gene expression of inflammatory factors, viz. tumor necrosis factor (TNF)‐α and IL‐6 as well as fibrosis‐related factors, that is, collagen‐I, TGF‐β, in skin, kidney, and lung tissues (Fig. 4B). The mRNA expression of TNF‐α showed tendency of elevation after the induction of SSc. IL‐6 was significantly increased especially in the kidney. TGF‐β mRNA expression also had trend of elevation similar to serum concentration. Expression of collagen‐I was found to increase significantly, especially in lung.

### TGF‐β/Smad‐3 signal pathway related to fibrosis in Scl‐GvHD mouse model

**Figure 5:**
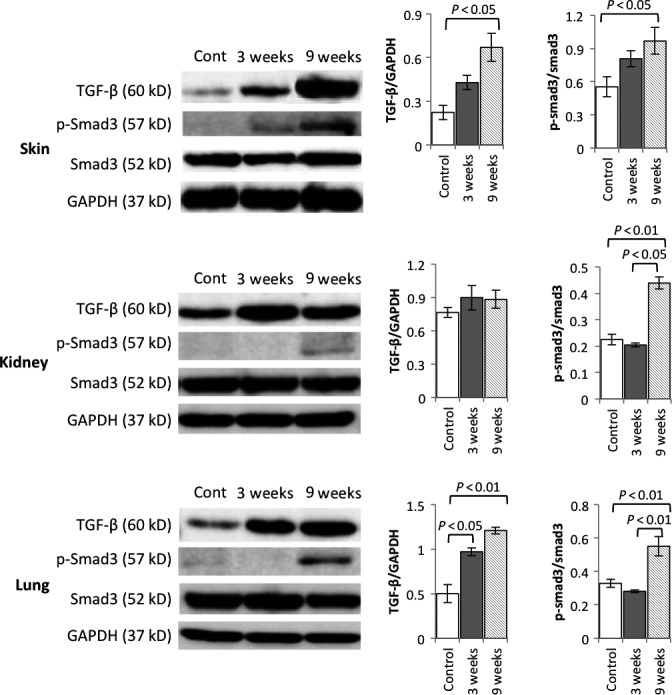
Expression of TGF‐β and phosphorylated Smad‐3 in scl‐GvHD mice. TGF‐β, Smad‐3, and p‐Smad‐3 protein expression in skin, kidney, and lung of scl‐GvHD mice after splenocytes transplantation of 3 and 9 weeks were detected by a western blot analysis. Data are the means ± SEM (*n* = 3–5). *P* < 0.05, *P* < 0.01 compared with the corresponding value of the control.

As TGF‐β is a central player in fibrosis, we examined the expression of the TGF‐β and phosphorylated Smad‐3 that is signaling molecule of TGF‐β in the tissue. We found that TGF‐β was significantly overexpressed in the skin and lung, with a tendency of higher expression in kidney of scl‐GvHD mice (Fig. 5). In addition, significant Smad‐3 phosphorylation could be detected in the skin, kidney, and lung of scl‐GvHD mice, and Smad‐3 phosphorylation increased with disease progression (Fig. 5). These data suggested that TGF‐β/Smad‐3 signal pathway plays a key role in the fibrosis of scl‐GvHD mice.

## Discussion

In this study, we demonstrated that intravenous injection of spleen cells from B10.D2 donor mice into immunodeficient Rag‐2 recipient mice on the BALB/c genetic background could induce severe fibrosis of the skin and internal organs such as the kidney and lung. Anti‐Scl‐70 antibody is known as a hallmark antibody of diffuse systemic scleroderma, and anti‐Scl‐70 antibody is associated with more severe scleroderma disease 1, 17. In our study, anti‐Scl‐70 antibody could be detected and increased with the progress of disease activity in scl‐GvHD mice model. Traditional scl‐GVHD model indicates lung fibrosis involvement. Our data proved that obvious lung fibrosis could be detected in the modified scl‐GvHD model. Severe fibrosis of the skin and internal organs, including kidney and lung, could be detected in our scl‐GvHD mice model, which is different from the previous scl‐GvHD model established by Ruzek *et al*. 18, which was characterized by severe fibrosis of the skin and internal organs, but excluding the lung.

Overproduction of collagen by fibroblast is a key feature of SSc; increased synthesis of collagen indicated fibroblast activation. Excessive collagen deposition and microvascular injury were pathologic hallmarks of SSc. However, the most of mechanisms of these pathogenic changes remain unclear.

Long‐standing observation of several types of lymphocytes infiltration into the skin and lungs of patients with SSc suggested that SSc is an immune‐mediated disease 19, 20. Early infiltration of mononuclear cells that are primarily of T lymphocytes and macrophages into the skin has been reported 20. Furthermore, the degree and progression of skin thickening were well correlated with the extent of mononuclear cell infiltration in the skin of patients with SSc 20. As consistent with previous study, our data also showed that increased infiltration of CD4^+^ T cells and F4/80^+^ macrophages could be detected in back skin, ear, kidney, and lung with the progress of disease course. These results indicate the role of inflammatory cells in the etiology of SSc on the top of the diverse mechanisms in which it may be an important pathogenetic factor for the disease. Although the role of these cells in the course of fibrosis in SSc is not clearly understood, inflammatory cytokines produced by these inflammatory cells can induce fibroblast collagen production and fibrosis.

Consistent with this speculation, TGF‐β and IL‐10, two strong profibrotic factors, were highly expressed in serum and in tissues of skin, kidney, and lung in the scl‐GvHD model. Furthermore, collagen deposition was observed in skin, kidney, and lung, and mRNA expression of collagen‐I also elevated in scl‐GVHD model. In accordance, the number of cells expressed α‐SMA, which was known to express extracellular matrix including collagen‐I, was increased.

TGF‐β and TNF‐α are associated with collagen synthesis and deposition, vascular change, inflammatory factors infiltration, and are also involved in the pathogenesis. In particular, TGF‐β is a central player in fibrosis, serving to the influx and activation of inflammatory cells, and an elaboration of extracellular matrix followed endothelial–mesenchymal transition and the influx of fibroblasts 21, 22. In addition to other models of fibrosis 23, TGF‐β works as a massive irritation to elevated collagen synthesis, which is assumed to be definitive in the pulmonary and cutaneous fibrosis in scleroderma 24. TGF‐β is secreted by several types of cells such as endothelial cells, fibroblasts, and monocytes and can be switched to the active form by matrix metalloproteinases expressed by monocytes/macrophages. TGF‐β/Smad‐3 pathway is a key pathway for the activation of fibroblast and collagen production 25, 26. In our scl‐GVHD model, elevation of serum and tissue TGF‐β expression along with mRNA expression of TGF‐β in tissue was observed. Furthermore, Smad‐3 is a key downstream signal protein of TGF‐β 25. We found that obvious Smad‐3 phosphorylation could be detected in skin, kidney, and lung of the scl‐GvHD model, which indicated that the TGF‐β/Smad‐3 pathway played a key role in the pathogenesis of the scl‐GvHD model.

IL‐10 acts as an anti‐inflammatory cytokine, which is expressed by monocytes and lymphocytes. Some study showed that IL‐10 might play important role in SSc susceptibility 27, 28. In our scl‐GVHD model, IL‐10 secretion was significantly increased. Furthermore, TNF‐α and IL‐6 act as strong pro‐inflammatory cytokines, also highly expressed in skin, kidney, and lung. Increased expression of these pro‐inflammatory cytokines can further induce fibroblast activation, collagen synthesis, and fibrosis. Thus, blocking these pro‐inflammatory cytokines might be a promising therapy for the treatment of SSc.

Scl‐GvHD mice showed generalized inflammation and tissue fibrosis that closely resembled the inflammatory phase of diffuse SSc. Scl‐GvHD model using Rag‐2‐KO mice is suitable for SSc research rather than traditional model, as radiation before transplantation is unnecessary. In Ruzek *et al*.'s study, however, there was little evidence of lung fibrosis compared with traditional scl‐GvHD using radiation 8, 18, 29. As reviewed by Greenblatt and Aliprantis 29, one possible explanation is that inflammatory ‘danger’ signals, such as those elicited by radiation injury, are important for the induction of a program that elicited fibrosis and inflammatory in the scl‐GvHD lung. However, a significant proportion of patients with SSc have fibrosis of the heart, kidney, gastrointestinal tract, and lung 30, 31, 32. Furthermore, several known pulmonary manifestations have been reported in each subset of the disease 33 and, in fact, interstitial lung disease and pulmonary hypertension may occur more commonly in a SSc 34. Furthermore, pulmonary disease can occur even in SSc with no skin involvement (an entity known as scleroderma sine scleroderma) 35. Therefore, pathological change in lung is one of the important symptoms of SSc.

To improve the model to exhibit all major aspects of clinical characteristics of SSc, we used Rag‐2‐KO mouse from different vendors and substrains. Our donor strain in this study was B10.D2/nSnSlc from SLC, Inc, in Japan, while a previous study used B10.D2‐H2^d^H2‐T18^C^HC^1^/nSnJ from Jackson Laboratories in the USA. Furthermore, the background of Rag‐2^−/−^ recipient is ‘BALB/cAJcl’ from CLEA Japan Inc. (Tokyo, Japan), whereas former study used same Rag‐2^−/−^ but with BALB/cAnNTac background (C.129S6 [B6]‐Rag‐2^tm1N12^) obtained from Taconic Laboratories (Germantown, NY, USA).

BALB/c mice are an inbred strain and distributed globally, resulting in them being one of the most widely used inbred strains. Halsey J. Bagg originally established this strain as the ‘Bagg albino’ at the Memorial Hospital, New York, in 1913. George Davis Snell, who was a student of Hermann Joseph Muller, named the line BALB/c in 1932 36. Andervont and Friis bred the BALB/c mice independently and established BALB/cAn and BALB/cA, respectively. BALB/cAnN was developed from the BALB/cAn line by the National Institutes of Health 36.

Substrains have only minor genetic differences; however in certain kinds of situation, even the same strain from distinct animal breeders can also show major differences with respect to some phenotypes 37. Several studies demonstrated that the phenotype was different among substrains. Coletti *et al*. 38 showed that BALB/cAnNCrl, BALB/cByJ, and BALB/cJ display major differences in physical activity as a behavior. Ohta *et al*. 39 showed the substrain‐dependent sperm abnormalities in BALB/c mice using BALB/cByJ, BALB/cA, and BALB/cAnN. The BALB/cAnNHsd had lower toxicity and better survival and was more resistant to developing glucocorticoid‐induced osteonecrosis as compared with BALB/cJ 40. Furthermore, BALB/cByJ mice showed more continual and severe dystrophic cardiac calcinosis than do BALB/cJ and BALB/cAnTac 41. In the field of immunity and infection, the control of growth of Taenia crassiceps between BALB/cAnN and BALB/cJ substrains was different 42, 43. Furthermore, BALB/cJ mice were resistant to the induction of both experimental allergic encephalomyelitis and experimental allergic orchitis, whereas BALB/cByJ mice exhibited a nonfully penetrant, susceptible phenotype 44. In addition, even though among the same mouse strain, mice from different breeding colonies showed different immune profiles among them. Ivanov *et al*. demonstrated that CB57BL/6 mice from Jackson Laboratory had few Th17 cells in lamina propria of the small intestine, whereas CB57BL/6 mice from Taconic had significantly higher number of Th17 cells in lamina propria of the small intestine. It was caused by the difference in microbiota between the mice. 45. Potter and Wax 46 demonstrated that 20% of pristane‐treated BALB/cAn mice and 70% of pristane‐treated BALB/cJ mice develop arthritis, which was known to be T cell‐dependent onset 47. This study suggested that T cells from BALB/cJ mice have higher reactivity to self‐antigen than those from BALB/cAn mice. Furthermore, it is hypothesized that monocyte activation by host‐reactive T cells is an initiating event in scleroderma and human sclerodermatous chronic GvHD. Infiltrating monocytes in the skin produce TGF‐β, resulting in upregulation of Smad‐3 phosphorylation and collagen and leading to fibrosis 48, 49.

Taken together, our data have proved that transplantation of spleen cells into immunodeficient Rag‐2 recipients on the BALB/c background from B10.D2 donor mice could induce severe fibrosis of the skin and internal organs, including kidney and especially in lung, which ensures us to understand the etiology and pathophysiology of SSc based on the modeling.

## Author contributions

XY and CL performed experiment. JY, XKL, and HZ designed experiment. CL, MF, and XKL analyzed data. CL, MF, and XKL wrote the manuscript.
